# Promoter methylation and large intragenic rearrangements of *DPYD *are not implicated in severe toxicity to 5-fluorouracil-based chemotherapy in gastrointestinal cancer patients

**DOI:** 10.1186/1471-2407-10-470

**Published:** 2010-09-01

**Authors:** Joana Savva-Bordalo, João Ramalho-Carvalho, Manuela Pinheiro, Vera L Costa, Ângelo Rodrigues, Paula C Dias, Isabel Veiga, Manuela Machado, Manuel R Teixeira, Rui Henrique, Carmen Jerónimo

**Affiliations:** 1 Department of Medical Oncology, Portuguese Oncology Institute - Porto, Portugal; 2 Department of Genetics, Portuguese Oncology Institute - Porto, Portugal; 3 Department of Pathology, Portuguese Oncology Institute - Porto, Portugal; 4 Cancer Epigenetics Group, Research Center of the Portuguese Oncology Institute - Porto, Portugal; 5 Cancer Genetics Group, Research Center of the Portuguese Oncology Institute - Porto, Portugal; 6 Department of Pathology and Molecular Immunology, Institute of Biomedical Sciences Abel Salazar, University of Porto, Porto, Portugal

## Abstract

**Background:**

Severe toxicity to 5-fluorouracil (5-FU) based chemotherapy in gastrointestinal cancer has been associated with constitutional genetic alterations of the dihydropyrimidine dehydrogenase gene (*DPYD*).

**Methods:**

In this study, we evaluated *DPYD *promoter methylation through quantitative methylation-specific PCR and screened *DPYD *for large intragenic rearrangements in peripheral blood from 45 patients with gastrointestinal cancers who developed severe 5-FU toxicity. *DPYD *promoter methylation was also assessed in tumor tissue from 29 patients

**Results:**

Two cases with the IVS14+1G > A exon 14 skipping mutation (c.1905+1G > A), and one case carrying the 1845 G > T missense mutation (c.1845G > T) in the DPYD gene were identified. However, *DPYD *promoter methylation and large *DPYD *intragenic rearrangements were absent in all cases analyzed.

**Conclusions:**

Our results indicate that *DPYD *promoter methylation and large intragenic rearrangements do not contribute significantly to the development of 5-FU severe toxicity in gastrointestinal cancer patients, supporting the need for additional studies on the mechanisms underlying genetic susceptibility to severe 5-FU toxicity.

## Background

The fluoropyrimidine 5-fluorouracil (5-FU) is broadly used in the treatment of a wide range of gastrointestinal cancers[1]. 5-FU is an analogue of uracil with a fluorine atom at the C-5 position replacing hydrogen, which enters the cell using the same transport machinery as the uracil nucleotide[2]. The mechanism of 5-FU cytotoxicity has been ascribed to the inhibition of thymidylate synthase (TYMS) and misincorporation of fluoronucleotides into RNA and DNA [1,3]. The rate-limiting enzyme in 5-FU catabolism is dihydropyrimidine dehydrogenase (DPD), which converts 5-FU to dihydrofluorouracil. More than 80% of administered 5-FU is normally catabolized primarily in the liver, where DPD is abundantly expressed[1].

The types of toxicity associated with 5-FU are predominantly myelosuppression, diarrhea, mucositis, and hand-foot syndrome[4,5]. The mechanism of 5-FU cytotoxicity may depend on genetic and clinical factors. Female gender and mode of administration in bolus are linked to increased toxicity[6]. In 5-FU bolus schedules, the incorporation of fluorouridine triphosphate into RNA appears to be the most important mechanism of action, whereas when the infusion time is prolonged, inhibition of TYMS becomes more important resulting in lower toxicity[7].

The expression levels of DPD and TYMS vary among individuals and may be related to different toxicity profiles. These might also be potentially important as prognostic factors and predictive markers of response to 5-FU[8,9]. However, no reliable molecular marker of sensitivity, resistance, or toxicity to 5-FU has been validated until now[10]. However, partial or complete DPD deficiency is a well known pharmacogenetic syndrome, detected in 3% to 5% of the general population, which has been associated with severe and potentially lethal toxicity following 5-FU administration[11].

The dihydropyrimidine dehydrogenase gene (*DPYD*), which codes for DPD, is present as a single copy gene on chromosome 1p22 and consists of 23 exons. Although more than 50 gene alterations have been characterized during the past decade[12], the majority of them represents missense or intronic variants with unknown biological and clinical significance[12,13]. Indeed, only a limited number of patients are carriers of allelic variants (including the most prevalent exon 14 skipping mutation, IVS14+1G > A) that significantly affect DPD catalytic activity[14]. Recently, large intragenic rearrangements of *DPYD *and a new interstitial deletion [del(1)(p13.3p21.3)] were found in some DPD deficient patients[15].

Nevertheless, the genetic variants reported thus far do not account for most of the DPD deficiency cases. Thus, epigenetic de-regulation of *DPYD *was hypothesized as an alternative mechanism for reduced DPD activity. In this setting, Noguchi and co-workers found that DPD activity was controlled at the transcriptional level by promoter methylation and thus aberrant methylation might affect the sensitivity to 5-FU in hepatocarcinoma cell lines[16]. Subsequently, Ezzeldin et al. assessed DPD activity and *DPYD *promoter methylation status in a small series of clinical samples (n = 15) from normal individuals and cancer patients [17]. *DPYD *promoter methylation was detected in peripheral bloods samples from all (five) DPD-deficient volunteers and in three out of five DPD-deficient cancer patients with a previous history of 5-FU toxicity. Interestingly, no evidence of methylation was detected in samples from five volunteers with normal DPD activity[17]. Finally, methylation of *DPYD *promoter region of RKO colorectal cancer cell line was shown to be associated with decreased gene expression[18].

We sought to characterize the *DPYD *promoter methylation status and the presence of large intragenic rearrangements in a series of gastro-intestinal (GI) cancer patients to determine whether these might constitute alternative mechanisms for DPD deficiency and a cause of severe 5-FU toxicity.

## Methods

### Study design

The primary objective of this cross-sectional study was to analyze the methylation status of *DPYD *promoter region in GI patients who developed severe 5-FU toxicity. The secondary objective was the analysis of large intragenic rearrangements of *DPYD*.

### Patient selection and Clinical evaluation

Forty-five patients with esophageal, gastric or colorectal cancer (Table 1) who had developed severe toxicity following chemotherapy regimens based on 5-FU and had been tested for *DPYD *exon 14 mutations (including the exon 14 skipping mutation IVS14+1G > A) by direct sequencing analysis at the Portuguese Oncology Institute - Porto, Portugal, from January 1994 through December 2008. All the patients were enrolled in this study, following informed consent. These studies were approved by the respective institutional review board (Comissão de Ética do IPO-Porto).

**Table 1:** Clinical and pathological characteristics of patient population

		n (%)
**No. of patients**		**45 (100)**
Gender; Median age (Min - Max), yrs	Female	24 (53.3); 54 (34 - 76)
	Male	21 (46.7); 61 (35 - 75)
ECOG	0	20 (44.4)
	≥ 1	25 (55.6)
Tumor Location	Esophageal	4 (8.9)
	Gastric	19 (42.2)
	Colorectal	22 (48.9)
TNM Stage	II	3 (6.7)
	III	12 (26.7)
	IV	30 (66.7)
Treatment Purpose	Adjuvant	14 (31.1)
	Palliative	31 (68.9)
Chemotherapy Scheme	5-FU/Cisplatinum	23 (51.1)
	Other*	22 (48.9)
5FU Mode of Administration	Bolus alone	15 (33.3)
	Bolus + CI^#^	8 (17.8)
	Continuous infusion	22 (48.9)

*5-FU/LV or 5-FU/FA or FolFOx or FolFIri; ^#^Continous Infusion

Data on patient demographics (gender and age), ECOG, tumor anatomical site, TNM staging, 5-FU-based chemotherapy scheme, mode of administration and toxicity profile were assessed by detailed hospital chart review for each case (Table 1). This study included 45 patients (24 women and 21 men). The median age was 56 years (ranging from 34 to 76 years) without differences across gender (*p *= 0.432). The combination of 5-FU with cisplatinum was given in 23 cases (51.1%), which were all esophageal or gastric cancer patients. In the 22 colorectal patients, 5-FU was given in conjunction with levamisole in 2 cases, combined with folinic acid in 12 cases. FolFOx was administered in 6 cases and FolFIri in 2 cases.

**Figure 1:**
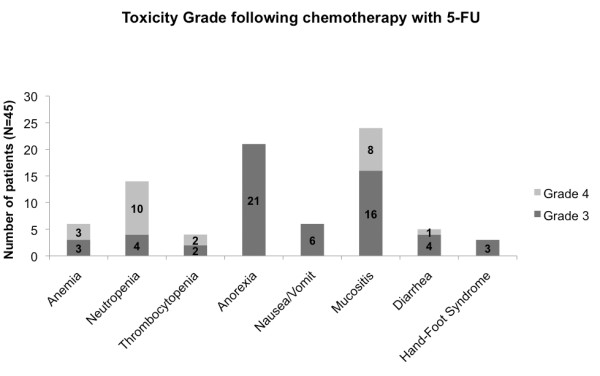
**Toxicity profile of the 45 patients with severe toxicity treated with 5-FU according to Common Terminology Criteria for Adverse Events, v4.0**.

Adverse drug effect during chemotherapy was classified according to the Common Terminology Criteria for Adverse Events (CTCAE) v4.0[19]. Accordingly, toxicity grades 3 or 4 were considered severe. Patients developed the following manifestations of toxicity grade 3 or 4 during chemotherapy with 5-FU (Figure 1): mucositis (24 cases, 53%), anorexia (21 cases, 47%), neutropenia (14 cases, 31%), anemia (6 cases, 13%), nausea/vomiting (6 cases, 13%), diarrhea (5 cases, 11%), thrombocytopenia (4 cases, 9%) and hand-foot syndrome (3 cases, 7%).

The clinicopathological characteristics and toxicity profile of three carriers of *DPYD *mutations identified in this study are provided in Table 2.

**Table 2:** Clinicopathological characteristics and toxicity profile of *DPYD *mutation carriers

	Patient #1	Patient #2	Patient #3
***Age***	35	64	68
***Gender***	female	male	female
***Tumor***			
*Location*	Colorectal	Colorectal	Esophageal
*Type*	Adenocarcinoma	Adenocarcinoma	Squamous cell carcinoma
*Stage*	IV	IIIB	IV
***Treatment regimen***			
***Toxicity profile***			
*Mucositis*	G3	G4	G4
*Anemia*	G4	G0	G0
*Neutropenia*	G4	G4	G0
*Thrombocytopenia*	G4	G0	G0
***DPYD mutation type***	1845 G > T	IVS14+1G > A	IVS14+1G > A

### Sample collection and tissue processing

A peripheral blood sample was obtained for all patients, and in 29 of these, a tumor-representative formalin-fixed, paraffin-embedded tissue block (biopsy or surgical specimen before chemotherapy) was available. Routine histopathological evaluation, comprising diagnosis, grading and pathological staging according to WHO[20] was performed in all cases. Serial sections were obtained from each of the representative tissue block and an expert pathologist delineated their highest proportion of malignant cells. Colorectal carcinoma cell line RKO from American Type Tissue Collection (ATTC, USA) was used as a positive control for methylation analysis (this cell line is methylated at the *DPYD *promoter). RKO cells were grown in ATCC-formulated Dulbecco's Modified Eagle Medium.

### DNA extraction, quantification and modification

DNA was extracted from all available samples (RKO cell line, peripheral blood and microdissected tumor tissue) with phenol-chloroform method[21] and quantified using Nanodrop™ ND1000 microspectrophotometer (NanoDrop, USA). Genomic DNA extracted from peripheral blood, cell line and microdissected tumor was submitted to sodium bisulfite conversion using a previously described method [22-24]. Briefly, 1 μg of genomic DNA from each sample were used for the chemical treatment. Bisulfite-modified DNA was purified using a vacuum manifold and a Wizard DNA Clean-up System [Promega Corp., WI, USA ], treated again with sodium hydroxide, precipitated with ethanol, eluted in 60 μl of water and stored at -80°C.

### Real-time, quantitative, methylation-specific PCR

The chemically modified DNA from the RKO cell line, peripheral blood and microdissected tumor tissue was amplified through quantitative methylation-specific PCR (QMSP) [25]. Primers were designed according to the CpG island at the promoter and exon 1 of the sense strand of the *DPYD *gene, starting at -266 bp from transcription start site (Genbank accession no. NM_000110) as follows: forward, 5'-TTTGTTTGTTTTCGATTCGC-3'; and reverse 5'-ATCCGCCGAATCCTTACTAA-3' (amplicon size of 208 bp). A reference gene (*ACTB*) was used to normalize for DNA input in each sample[26]. Fluorescence based QMSP assays were carried out using the SYBR^® ^Green PCR Master Mix (Applied Biosystems, USA). Running conditions were: 50°C for 2 min, 95°C for 10 min followed by 45 cycles of 95°C for 15 sec, and 60°C for 1 min. After 45 cycles, a dissociation-curve analysis was performed using the following conditions: 95°C for 15 sec, 60°C for 20 sec and 95°C for 30 sec. Each sample was run in triplicate and, additionally, multiple water blanks were used per plate as a control for contamination (negative control). All amplifications were carried out in 96-well plates on a 7500 Sequence Detection System (Applied Biosystems, USA). In each plate, five serial 10x-dilutions of fully methylated, bisulfite converted DNA - CpGenome Universal Methylated DNA [Millipore, CA, USA] - were also included to construct the standard curve in order to quantify the amount of fully methylated alleles in each reaction. A run was considered valid when the following six criteria were met: (1) slopes of each standard curve above -3.60 corresponding to a PCR efficiency > 90%; (2) R^2 ^of at least four relevant data points ≥ 0.99; (3) no template controls not amplified; (4) the positive methylation control had to provide a methylated signal; (5) the negative control had no signal; and (6) threshold cycle value for each gene ≤ 40. To determine the relative levels of methylated promoter DNA in each sample, the values obtained by QMSP analysis (mean quantity) for the target gene were divided by the respective values of the internal reference gene (*ACTB*). The ratio thus generated, which constitutes an index of the percentage of input copies of DNA that are fully methylated at the primer site, was then multiplied by 1000 for easier tabulation (methylation level = target gene/reference gene × 1000).

### *DPYD *large genomic rearrangements analysis

Peripheral blood DNA samples were also screened for *DPYD *large genomic rearrangements by Multiplex Ligation Probe Amplification (MLPA) according to the manufacturer's instructions (SALSA P103 kit; MRC, Holland). This probemix contains probes with target-specific sequences for each of the 23 exons and promoter region. Owing to the large size of the introns, probes for five of the introns are also included. The MLPA method is based on sequence-specific probe hybridization to genomic DNA, followed by PCR amplification of the hybridized probe (with one FAM-labeled primer) and semi-quantitative analysis of the PCR products. A deletion or duplication was scored if the relative peak area of the amplification product presented a reduced or augmented area of 35 to 50% when compared to a normal control, respectively. The kit also contains a probe specific for the exon 14 skipping mutation (IVS14+1G > A) that only generates a signal when the mutation is present [27].

### Statistical analysis

Descriptive statistics were used to summarize the clinicopathological, molecular and immunoexpression data. The independent samples Mann-Whitney U test was applied to determine if the age distribution was the same across genders. The analysis was performed with PASW Statistics 18.0 software.

## Results

**Figure 2:**
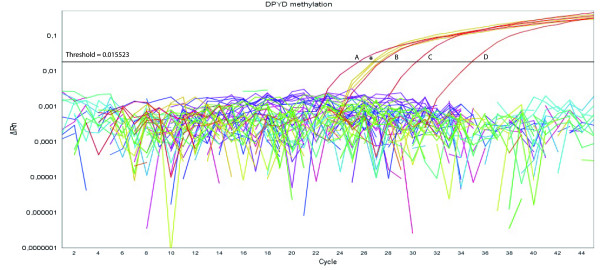
Illustrative QMSP amplification plots for 4 serial 10×-dilutions of fully methylated, bisulfite converted DNA (A, B, C, D) and *DPYD *promoter methylation in RKO cell line (*). The *DPYD*/*ACTB *ratios were determined using the cycle number where fluorescence per reaction crossed the threshold, which is set to the geometrical phase of polymerase chain reaction amplification above background. ΔRn is defined as the cycle-to-cycle change in the fluorescence signal (log scale).

No evidence of *DPYD *promoter methylation was observed in any of the 45 peripheral blood samples nor in the 29 microdissected tumor tissue samples from patients experiencing severe 5-FU toxicity. Nevertheless, *DPYD *promoter methylation was detected in the RKO cell line (methylation ratio = 1075) (Figure 2).

**Figure 3:**
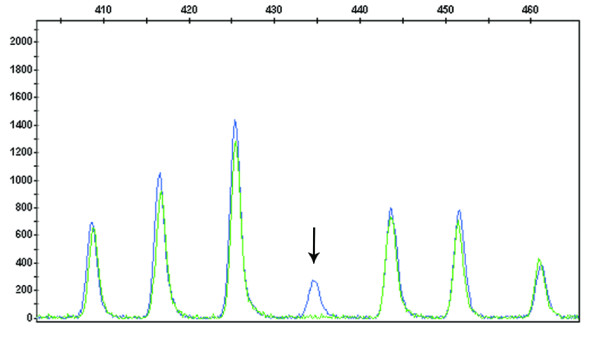
**Capillary electrophoresis pattern of one normal control (green) and one case presenting the IVS14+1G > A mutation in the *DPYD *gene (blue) detected by MLPA analysis**. *DPYD *MLPA probe-mix presents a probe specific for the IVS14+1G > A mutation that will only generate a signal when the mutation is present (arrow).

Concerning mutation analysis, the MLPA assay was performed in 42 of 45 cases (3 cases were not studied due to low quality DNA). We were able to identify (Figure 3) the two exon 14 skipping mutation (IVS14+1G > A) previously detected by direct sequencing analysis, but not the 1845 G > T missense mutation. The remaining 39 patients with severe toxicity to 5-FU treatment that did not carry clinically relevant allelic variants in exon 14 of *DPYD*, were further screened for large *DPYD *intragenic rearrangements. No intragenic rearrangements were found for *DPYD *in the peripheral blood samples of those patients.

## Discussion and Conclusions

5-FU is broadly used in the treatment of GI cancer. Deficiency of the enzyme DPD, encoded by the *DPYD *gene, has been associated with the development of severe toxicity to 5-FU in GI cancer patients. Since aberrant promoter methylation has been proposed as an alternative mechanism of DPD deficiency, we assessed the methylation status of *DPYD *promoter region by quantitative methylation-specific PCR in GI cancer patients who developed 5-FU-related severe toxicity. Moreover, the analysis of large intragenic rearrangements of *DPYD*, which have also been causally related with DPD deficiency, was additionally performed.

Severe toxicity associated with 5-FU has been attributed in a small number of cases to allelic variants of the *DPYD *gene, which result in lower DPD enzyme activity. However, in most cases of severe 5-FU toxicity no genetic mechanism has been described. Thus, a possible role for epigenetic alteration of *DPYD*, especially promoter methylation, has been hypothesized. The first published study on this issue found that methylation of *DPYD *promoter in peripheral blood leucocyte DNA from colorectal patients was associated with severe 5-FU toxicity[17]. In our series of 45 patients with GI cancer with severe 5-FU toxicity, methylation at the *DPYD *promoter was not found in any of the cases, neither in peripheral blood leucocytes nor in tumor tissue samples. Importantly, a well characterized colon cancer cell line (RKO), known to harbor extensive CpG methylation at the *DPYD *promoter, tested positive, thus validating the methodology used in our study. Indeed, while this study was being performed, two independent studies reported similar findings in peripheral blood leucocytes of 28 patients[6] and 17 patients[28] with severe toxicity following 5-FU administration. It is noteworthy that the first cited study examined only 15 individuals, of which only five were cancer patients[17], whereas the two latter studies[6,28] and our own comprise a total of 90 patients, Indeed, our study has the largest single series of patients among all cited studies. Thus, our data further sustains that *DPYD *promoter methylation is absent in patients treated with 5-FU for GI cancer which have developed severe toxicity.

All patients enrolled in this study were screened for *DPYD *exon 14 mutations by sequencing (including the exon 14 skipping mutation IVS14+1G > A), eight of which were included in a previous publication on unselected colorectal cancer patients[29]. This mutation was found in two patients (one previously reported) and both of them developed severe toxicity following 5-FU administration. Previous observations confirmed the high specificity, positive and negative predictive values of this genetic analysis[6,14,30]. However, our previous analysis of 73 consecutive colorectal cancer patients detected exon 14 mutations in only two of eight cases with severe toxicity[29].

Both patients harboring the skipping mutation in exon 14 (IVS14+1G > A) and the patient harboring the 1845 G > T missense mutation, suffered from grade 4 toxicity (mucositis and febril neutropenia) related to 5-FU chemotherapy [29]. Thus, although DPYD mutational screening identifies cases which are prone to develop 5-FU-related toxicity, the low prevalence of those mutations might raise questions regarding the cost-effectiveness of the procedure.

Considering the results obtained for *DPYD *promoter methylation and skipping mutation in exon 14 (IVS14+1G > A) analysis, we decided to determine whether large *DPYD *intragenic rearrangements might explain 5-FU toxicity. However, those large intragenic rearrangements were also not found in any of the 39 cases, thus excluding a significant role for this genetic alteration in the impairment of DPD activity. This result is also in agreement with a recently published study in which no large rearrangements were found in series of 68 patients experiencing severe 5-FU toxicity[31,32].

In conclusion, considering our own and previously published data, epigenetic silencing and intragenic rearrangements of *DPYD *do not contribute to the development of severe 5-FU toxicity in GI cancer patients. Although severe 5-FU toxicity is a significant clinical concern, additional studies integrating a more comprehensive analysis of 5-FU metabolic pathway are required to uncover the factors underlying the majority of patients which experience severe 5-FU toxicity.

## Competing interests

The authors declare that they have no competing interests.

## Authors' contributions

JS-B collected the clinical data, carried out experiments and drafted the manuscript. JR-C, MP and VLC carried out experiments. AR assisted in the review of the histopathological material. PCD carried out experiments including tissue block sectioning. MM helped with the collection of clinical data. MRT was involved in drafting the manuscript. RH helped in the design of the study, performed the review of the histopathological material and assisted in the finalization of the manuscript. CJ designed and supervised the project, and finalized the manuscript. All authors have read and approved the final version of the manuscript.

## Pre-publication history

The pre-publication history for this paper can be accessed here:

http://www.biomedcentral.com/1471-2407/10/470/prepub
